# Current Knowledge and Future Perspectives on Awake Bruxism Assessment: Expert Consensus Recommendations

**DOI:** 10.3390/jcm11175083

**Published:** 2022-08-30

**Authors:** Alessandro Bracci, Frank Lobbezoo, Birgitta Häggman-Henrikson, Anna Colonna, Laura Nykänen, Matteo Pollis, Jari Ahlberg, Daniele Manfredini, International Network for Orofacial Pain and Related Disorders Methodology (INfORM)

**Affiliations:** 1 Department of Neurosciences, School of Dentistry, University of Padova, 35128 Padova, Italy; 2 Department of Orofacial Pain and Dysfunction, Academic Centre for Dentistry Amsterdam (ACTA), University of Amsterdam and Vrije Universiteit Amsterdam, 1081 LA Amsterdam, The Netherlands; 3 Department of Orofacial Pain and Jaw Function, Faculty of Odontology, Malmö University, 20506 Malmö, Sweden; 4 Department of Biomedical Technologies, School of Dentistry, University of Siena, 53100 Siena, Italy; 5 Department of Oral and Maxillofacial Diseases, University of Helsinki, 00100 Helsinki, Finland

**Keywords:** awake bruxism, bruxism, ecological momentary assessment, electromyography, masticatory muscle activity, self report, sleep bruxism, temporomandibular disorders

## Abstract

Awake bruxism (AB) is differentiated from sleep bruxism (SB) by the differences in etiology, comorbidities, and consequences related to the different spectrum of muscle activities exerted in relation to the different circadian manifestations. Furthermore, less literature data are available on AB than on SB. The introduction of ecological momentary assessment (EMA) strategies has allowed for collecting valuable data on the frequency of the different activities reported by an individual in his/her natural environment. This strategy has been further improved with the recent use of smartphone technologies. Recent studies have described an average frequency of AB behaviors, within the range of 23–40% for otherwise healthy young adults. An association between AB and some psychological traits has emerged, and the findings have indicated that patients with musculoskeletal symptoms (e.g., temporomandibular joint and/or muscle pain, muscle stiffness, and fatigue) report higher AB frequencies. Preliminary data suggest that muscle bracing and teeth contact are the most commonly reported behaviors, while teeth clenching is much less frequently reported than commonly believed previously. Report of teeth grinding during wakefulness is almost absent. This paper has critically reviewed the currently available approaches for the assessment of AB. In addition, some future perspectives and suggestions for further research have been provided.

## 1. Introduction

Among the conditions receiving increasing attention within the dental and medical communities, bruxism is an important topic because of its several clinical and research implications. In particular, while the focus of past studies on bruxism was on the potential dental consequences, such as tooth wear and prosthodontic complications, the last decade has been characterized by several updates and improvements on the topic of bruxism definition and evaluation [1]. Two expert consensus papers contributed substantially to define the construct of bruxism as an umbrella term to embrace a multifaceted spectrum of masticatory muscle activities, not limited to the act of grinding teeth while asleep. Within this framework, as an update with respect to the overall definition adopted in a 2013 paper [2], separate definitions have since 2018 been recommended for both sleep bruxism (SB) and awake bruxism (AB) [3].

Both definitions place emphasis on the role of masticatory muscle activity during sleep and wakefulness as the source of potential clinical consequences, as well as the fact that, while bruxism is not a disorder in most individuals, it could be a sign of a disorder in others. Tiredness and/or pain in the masticatory muscles and the temporomandibular joint (TMJ) and functional limitations, together with prosthodontic complications and mechanical tooth wear, are examples of potential negative health outcomes due to bruxism. On the other hand, the restoration of upper airways patency in some patients with sleep apnea and stimulation of brain activity in individuals with cognitive decline could be considered as examples of possible positive health outcomes [4,5,6].

In relation to the circadian rhythm, SB is a much more studied condition than AB [7,8]. SB is defined as a masticatory muscle activity during sleep that is characterized as rhythmic (phasic) or non-rhythmic (tonic), and is not a movement disorder or a sleep disorder in otherwise healthy individuals [3]. AB is defined as a masticatory muscle activity during wakefulness that is characterized by repetitive or sustained tooth contact and/or by bracing or thrusting of the mandible, and is not a movement disorder in otherwise healthy individuals [3]. From an etiological perspective, AB seems more directly associated with psychological traits [9,10,11], whereas SB is a complex activity with multiple neurological implications and interactions with other sleep-related conditions [12,13,14,15].

An important shortcoming of the bruxism literature is that, while assessment strategies on SB have often been discussed and evaluated since the early 1990s, few studies have been performed on the evaluation of AB [16,17,18]. Recently, a Standardized Tool for the Assessment of Bruxism (STAB) has been proposed and is currently undergoing refinement by an expert panel [19]. One of the concerns that emerged from the works of the expert consensus panel that contributed to the updated bruxism definition and assessment strategies [3,19] is that a summary of the available approaches to evaluate awake bruxism is actually missing.

Given the above premises regarding the need to study AB more in depth, the present paper aims to discuss the current knowledge on awake bruxism assessment and to suggest a future research agenda on the topic.

## 2. Assessment of Awake Bruxism Status

One of the main challenges that led to the development of the Standardized Tool for the Assessment of Bruxism (STAB) was “how to assess an individual’s bruxism in a reliable, valid, and relevant way?”. The word “relevant” assumes importance because of its implication that, in addition to the presence or absence of masticatory muscle activity, clinicians should be able to determine the point at which bruxism is likely to be associated with clinical consequences.

The 2013 consensus paper suggested a diagnostic grading system for possible, probable, and definite sleep/awake bruxism, but it is now clear that current knowledge and strategies are not sufficient to pursue such a hierarchical approach to evaluation [2,3,16]. Based on this, approaches for assessing bruxism can be distinguished as non-instrumental or instrumental [7,19]. A brief review with a focus on the implications for AB will be provided here.

In general terms, non-instrumental approaches for assessing bruxism include self-report (questionnaires and oral history) and clinical examination [3,20], which can be used both for AB and SB. Nonetheless, some general remarks should be made concerning their potential usefulness. Firstly, it must be kept in mind that clinical assessment is actually deemed to evaluate the presence of the purported consequences of bruxism, rather than the bruxism status itself [21,22]. In addition to that, it must also be pointed out that clinical signs related to AB are hard, if not impossible, to distinguish from the consequences of SB. The only possible exception is intrinsic mechanical tooth wear (i.e., attrition-related), which is unlikely to be related to AB, as teeth grinding rarely occurs during wakefulness [23,24]. The same consideration can be made for any prosthetic complications, given the fact that awake bruxism is mostly exerted in the form of mandible bracing without teeth contact. As for self-reported data, the validity of sleep-related findings is questionable for obvious reasons related to the unconsciousness of patients during sleep [25].

Self-report via structured questionnaires and interviews may be useful to gather information on perceived bruxism activities and their possible associated factors [26,27,28,29]. However, as pointed out in the 2018 consensus paper, the intensity and duration of specific masticatory muscle activities cannot be quantified via self-report [3]. In addition, one further limitation is that the bruxism−psyche relationship could alter self-reporting, reflecting either distress or a patient’s belief rather than masticatory muscle activity per se. Thus, the derived evaluation of bruxism status based on self-report is at risk of having limited value because of its subjectivity. Nonetheless, it represents the best available strategy to gather data for epidemiological purposes and to screen for the possible presence of bruxism at an individual level.

For the specific assessment of AB, there are currently no universally adopted dedicated questionnaires. The most frequently used approach in the research setting provides the use of awake bruxism items included in history taking instruments that were designed for broader scopes, such as the report of bruxism (e.g., Bruxscale) [30], temporomandibular disorders (e.g., Diagnostic Criteria for Temporomandibular Disorders (DC/TMD)) [31], or oral behaviors (e.g., Oral Behaviors Checklist) [32]. The time span and frequency in which the report of awake bruxism is referred varies among the different questionnaires.

**Figure 1:**
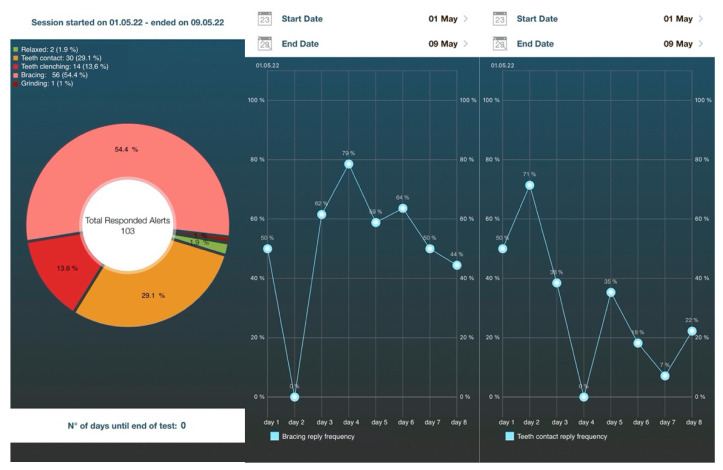
An example graphical interface to report the ecological momentary assessment of awake bruxism behaviors over one week or more. Percentages refer to the frequency of each specific answer over a one-week period and their trend day by day.

As a possible implementation of improved self-reported data collection, patients may be asked to monitor and report their behavior real time over a 1- or 2-week period after being informed of the possible conditions belonging to the spectrum of AB behaviors (i.e., clenching, bracing, thrusting, and teeth contact habit) [33]. Such an ecological momentary assessment (EMA) approach, also called experience sampling methodology (ESM), can improve the quality and quantity of data collection, as it provides multiple time-point reporting in real time over an observation period through the use of diaries or dedicated smartphone apps [34,35] (Figure 1). Several studies have recommend the use of such EMA strategies to report AB behaviors and to collect real-time reports on specific oral conditions that are related to the spectrum of AB activities, while also allowing for the assessment of the association with some possible etiological factors (e.g., psychosocial traits) and consequences (e.g., masticatory muscle pain) [36,37,38].

**Figure 2:**
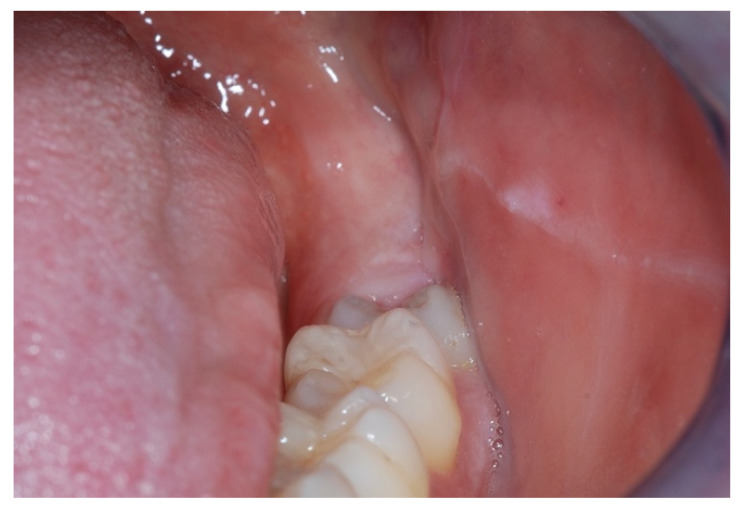
Severe linea alba of the cheek mucosae, which is a potential sign of prolonged bruxism activity in the form of bracing and/or clenching.

Concerning clinical examination, it should include an extraoral evaluation and an intraoral inspection to identify the signs and symptoms possibly related with bruxism [19,20]. The extraoral evaluation should assess the jaw muscles (e.g., evident muscle hypertrophy), the TMJ (e.g., presence of TMJ noises suggestive of disc displacement or joint degeneration), the presence of pain (e.g., jaw-muscle pain, TMJ pain, and headache), and functional symptoms (e.g., difficulty opening the mouth wide). The intraoral inspection should include a comprehensive dental examination (e.g., tooth wear, tooth enamel chipping, cracks and fractures of natural teeth, restoration failures, tooth mobility, and periodontal ligament widening on radiographic imaging) and an inspection of the mucosa of the cheek, lip, and tongue (e.g., linea alba, tongue scalloping, and traumatic lesions), as well as the presence of intraoral pain (e.g., teeth soreness and/or hypersensitivity) (Figure 2). As pointed out above, this approach is more oriented to identify the potential consequences of bruxism rather than the actual bruxism activity, and is unlikely to discriminate between AB and SB [39,40,41]. All clinical signs and symptoms should thus be assessed within the framework of a comprehensive differential diagnostic process.

**Figure 3:**
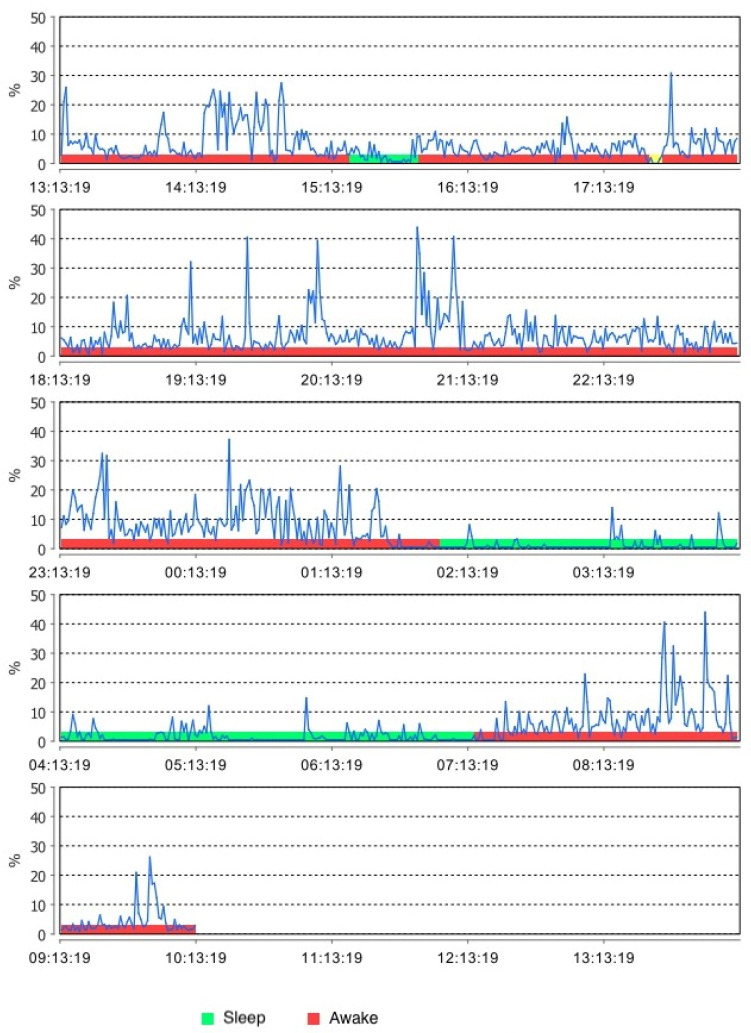
An example trace of electromyographic (EMG) recording of the masseter muscle by using a miniaturized device for in-home 24 h recordings.

Instrumental approaches, which have been available and used for years to record SB activities, can also be recommended for the evaluation of AB. EMG recordings during wakefulness may indeed provide measurements of AB, but such a strategy is currently not easy to perform due to the paucity of dedicated devices on the market. A feasible combination of features of hardware (e.g., electrode quality, long-duration battery, miniature size, and wireless functioning) and software (e.g., data analysis and graphical interface) is required [17]. Recent developments are promising, but on-field studies are yet to be performed on a larger scale, also with the aim of refining the discrimination between AB and non-AB activities during wakefulness (Figure 3).

From a conceptual viewpoint, the recent introduction of smartphone applications based on EMA principles also belongs to the category of instrumental approaches, even if data are gathered via self-report. After some early studies based on pagers, diaries, cell phones, or wrist clock alerts, this strategy has recently been optimized with the development of a user-friendly interface, opening up a new era for the EMA approach. This data-recording strategy can find applications for both research and clinical purposes. In the research setting, it allows for gathering a huge amount of data on the epidemiology of different AB behaviors at population levels, while at the individual level, in the clinical setting, it helps patients to recognize their habits, monitor changes over time, and adopt corrective measures [33].

## 3. Summary of the Available Literature

Data on the prevalence and natural course of bruxism are mostly derived from self-reported approaches and are mainly related to SB [42]. For AB, the reported prevalence rates drawn from questionnaire studies range from 22% to 30%, without any concrete and reliable reports about fluctuations over time. Furthermore, in 2013, a systematic review on the prevalence of bruxism in adult populations cautioned about the interpretation and generalization of findings due to the poor methodological quality of the reviewed literature, particularly regarding the amount of papers relying on single-item self-reports to assess bruxism [43].

Until recently, EMA-gathered data have been fragmental and limited to a few investigations on selected behaviors and populations, mainly reporting data collected with pagers or instruments and software developed for that specific investigation [44,45,46]. After the introduction of the updated bruxism definitions in 2018, concurrent assessment strategies have been developed. In particular, a smartphone-based approach, which was recently introduced to implement EMA in the clinical research setting, was dedicated to collecting data on the reported frequency of the conditions that are potentially part of the AB spectrum [47]. An early report provided data on the frequency of the above-described AB behaviors over a one-week time span in a sample of healthy young adults through the adoption of a dedicated smartphone application. The findings reported a 28.3% frequency of AB behaviors over one week of EMA monitoring, with a low coefficient of daily variation for the report of the relaxed jaw muscle condition [48].

More recently, several studies were performed that focused on two important aspects to refine this approach at the individual level, viz., compliance and comprehension. Based on the currently available data, patients’ compliance, defined as the percentage of responses in real time to received alerts, is over 60% [36]. The patients’ comprehension of reporting AB can be maximized by providing training to the clinician who prescribes the app, in order to put him/her in the condition of carefully explaining to the patients the meaning of the AB terms indicating the various behaviors [49].

A common approach to smartphone-based EMA investigations has been to report any of the possible AB behaviors over a seven-day observation period. As a general remark, in the field of scientific research on large populations, it must be noted that such an approach allows for the collection of a huge amount of data, with thousands of alerts answered with self-report of the condition in real time (up to 20 alerts multiplied for n participants multiplied for n days) [47]. Based on this, these findings are hard to compare with other studies due to the different study designs, as most studies are retrospective with the commonly used strategy being to collect self-reported data at single time points. The massive data collection is a feature of all EMA observational studies, and can provide normative values for EMA-based frequency of AB behaviors, as well as allow for investigation of associations with additional single-item reports on dietary or smoking habits, medication usage, psychological issues, and comorbid conditions. On the other hand, the large amount of data from EMA require careful management for statistical analysis purposes and it may paradoxically be difficult to interpret the findings.

So far, most available data have been collected in populations of healthy young students, who reported teeth contact as the most frequent behavior over seven days (13.6% in the largest study conducted so far) [50]. Interestingly, the fact that teeth grinding is rarely reported in any study populations from different countries is a promising finding to explore for discriminating AB from SB in terms of etiology, muscle behaviors, and possible consequences. From a dental perspective, this information is important for studying the role of bruxism in relation to tooth wear and implant complications, which are hardly viewed as a consequence of awake bruxism.

In general, smartphone-based EMA studies have suggested that the frequency of AB behaviors in young adults is within the 28.3–40% of alerts received over one week of EMA monitoring [11,37,38,48]. These findings could be seen as a reference point for future investigations on the epidemiological features of AB, as well as for comparison with selected populations of individuals with a purported higher prevalence of bruxism due to potential risk factors (e.g., psychological features) or observed consequences (e.g., muscle fatigue and muscle pain). As far as gender differences are concerned, a systematic review did not find any gender differences in the frequency of AB, but none of the included studies were based on EMA [43]. In EMA studies, the average reported frequency was higher in women than in men. Hence, differences in methodology may, at least partly, explain the differences in gender-related findings.

Concerning the fluctuations over time, it is interesting to note a very low coefficient of daily variation over one week for the non-AB (i.e., relaxed) muscle condition (0.27–0.44) [48,50]. This means that the frequency of AB behaviors as a whole in a population of healthy young adults does not change relevantly from one day to another in a short-time span. Indeed, the variability of reporting over a one-week span mostly concerns the type of specific behavior on the AB spectrum. The fact that the report of specific AB behaviors is quite variable may be explained by natural fluctuation, as well as some difficulties by the patient to recognize them consistently. Such findings are nonetheless important, because they suggest that the conditions may be well recognized by an individual. Based on the low day-to-day variability in the average frequency value, EMA strategies may be considered useful to collect reliable estimates of AB, thus reducing the influence of natural fluctuation related to the assessment of specific AB conditions. Future studies could be designed to assess the factors that are responsible for the observed variation in AB behaviors over prolonged periods of time. This goal fits with the current demand to support a “4 A” approach to the study of bruxism, which is accurate and reliable (valid), applicable (feasible), affordable (cost-effective), and accessible (suitable for everyday clinical use) [3].

Importantly, within the EMA framework, the concept of natural fluctuation and the course of AB behaviors is hardly distinguishable from a conditioning effect, viz., ecological momentary intervention (EMI), which may also offer interesting perspectives from a therapeutic viewpoint. For instance, all data collected so far showed a slight decrease in the average frequency of reported AB behaviors during a one-week assessment. This finding is in agreement with the theory that being asked about a behavior in close contextual and temporal proximity to its occurrence draws an individual’s attention towards the behavior, thereby promoting self-awareness and potentially inducing positive changes with respect to the capability to self-recognize and avoid it (i.e., EMI-biofeedback) [51,52,53].

Concerning EMG, although it represents the theoretical standard of reference for an evaluation of the full spectrum of activities included under the AB umbrella, there are currently no systematic data on the EMG features of muscle behaviors as recorded during wakefulness. Thus, suggestions on how to increase knowledge on this topic are provided in the section below.

## 4. Future Perspectives for Research Planning

Studies based on self-reported strategies for AB, and bruxism in general, have always been considered biased due to the purported questionable reliability and validity of this approach.

Concerning reliability, a recent study suggested that, once adequate information was given to the clinicians who prescribed the use of smartphone-EMA and to the patients who use it, no significant differences in reported AB prevalence were found between two university samples of healthy students [50]. In particular, identical instructions on how to use the application were provided to both study populations, with the same supporting educational materials (viz., slides, images, and videos) presented by the same investigators. In addition, the participants downloaded the very same version of the smartphone application, regardless of the model of smartphone they owned. This attempt to minimize the dishomogeneity of information might have been instrumental to “calibrate” self-reporting at individual and group levels. In addition, an ongoing investigation on the test−retest repeatability of reports confirmed the usefulness of adequate patient training to help them understand the intended meaning of the terms formulated to indicate the different AB conditions [49]. Thus, future studies might support the assumption that an approach based on carefully organized and standardized training sessions leads to a reliable EMA-based self-report. On the other hand, it must be underlined that the features of a study population might influence the results of all training efforts, as participants with different ages, educational levels, and socioeconomic statuses, and even concurrent health problems, may differ in response to educational sessions and in compliance with the observation protocols.

Concerning validity, information is actually lacking and opens the door for many potential developments for future EMA research. Validation studies should be performed to test the hypothesis that the strategy to report AB correlates with the actual muscle activity. The derived findings may be useful to refine the assessment strategies both for self-reported and instrumental strategies [3,19]. Investigations on the potentially associated factors (e.g., dietary or smoking habits, and medications usage) and conditions (e.g., psychological state, personality traits, and comorbid conditions) that may theoretically increase or decrease the frequency of AB behaviors may also be performed and have an impact on the clinical management of AB patients [1,53]. Cross-population comparisons to test for the existence of country and sociocultural differences are also possible by virtue of the multi-language platforms of the available smartphone applications. In addition to this, investigations on the potential advantages of using EMA with respect to less elaborated self-report strategies should be performed. Indeed, EMA requires compliance as well as basic technological equipment for smartphone-based strategies, which may represent a bias for the selection of the study population.

As for EMG recordings, given the complexity of this phenomenon, the construct of bruxism has been extended to include a wider spectrum of jaw-muscle activities than provided in the past [16]. This has important implications, especially for the interpretation of previous SB studies based on the count of masseter EMG events associated with sleep arousals, but also for investigations on AB. For instance, there are preliminary data collected with a novel EMG recording device that can be safely used to monitor masseter muscle activity over the full 24 h span that allows for a more comprehensive elaboration of the EMG signal in terms of muscle work and the bruxism time index [54]. Deeper probing into this area will allow for a better discrimination between AB and physiological activities (e.g., chewing, yawning, talking, and swallowing). For researchers, such an approach can be used to increase knowledge on several aspects of AB, including the natural course and fluctuations of signs and/or symptoms, the relationship with SB, and the exposure to etiological factors. Furthermore, a remarkable amount of data are going to be available to study the epidemiology of the various items at the individual (e.g., case series) as well as at the population level (e.g., cross-sectional and longitudinal large-sample studies). Such data might be used to standardize future reports for comparison purposes. As an important clinical implication, the data could also be useful for monitoring bruxism evolution over time, thus assisting clinicians in preventing and managing the possible consequences at an individual level.

Based on the above considerations, it has emerged that knowledge on AB epidemiology would benefit a lot from the adoption of clinically-oriented research studies aiming to identify it from a qualitative and quantitative viewpoint. A comprehensive approach including a combination of self-reported and measurement strategies, each of which may have some disadvantages that are compensated by the advantages of using it in combination with other approaches, will likely emerge as the gold standard for evaluating awake bruxism. These considerations are in line with the aims and strategies adopted in the ongoing STAB project.

## 5. Conclusions

Knowledge on bruxism is rapidly evolving. The most important evolution relates to the concept of bruxism itself, which is now viewed as an umbrella term for different jaw muscle activities, both during sleep and during wakefulness, that are not necessarily related to specific sleep correlates or to teeth contact. This means that future development in the approaches to evaluate AB must reflect the ongoing paradigm shift in the definition of bruxism. To this aim, adopting a combination of approaches, encompassing self-reported and instrumental evaluations, as being currently refined in STAB, is likely to emerge as the best possible strategy to overcome the limitations of the different stand-alone approaches.

## Data Availability

Not applicable.
